# Continuous Erector Spinae Plane Block in Paediatric VATS: A Case Series

**DOI:** 10.5152/TJAR.2023.21591

**Published:** 2023-02-01

**Authors:** Vishal Saxena, Harick Shah, Swarup Ray, Amrit Kaur, Raylene Dias

**Affiliations:** 1 Department of Anaesthesiology, Seth GS Medical College and KEM Hospital, Mumbai, India; 2 Department of Paediatric Anaesthesia, SRCC Children’s Hospital, Mumbai, India; 3 Department of Paediatric Anaesthesia, Seth GS Medical College and KEM Hospital, Mumbai, India

**Keywords:** Continuous erector spinae plane block, ESP block with catheter, paediatric anaesthesia, paediatric empyema, video-assisted thoracoscopic surgeries

## Abstract

Decortication and stripping of infected pleura by video-assisted thoracoscopic surgery or thoracotomy is the treatment of choice in cases of empyema. The stripping is associated with intense post-operative pain. Erector spinae block is an excellent and safe alternative to thoracic epidural block. The experience in paediatric erector spinae plane block is very limited. We present our experience of continuous erector spinae block and one single-shot erector spinae plane block in paediatric video-assisted thoracoscopic surgeries. We had 5 patients aged 2-8 years with right-sided empyema, who were taken up for video-assisted thoracoscopic surgery decortication, and 2 patients aged 1-4 years with congenital diaphragmatic hernia (CDH) for video-assisted thoracoscopic surgery CDH repair. After induction and intubation, using high-frequency straight ultrasound probe, an erector spinae plane catheter was inserted and the local anaesthetic agent was administered. The patients were monitored for signs of effective analgesia. Post-extubation continuous erector spinae plane block was continued for 48 hours using bupivacaine and fentanyl. All patients had excellent postoperative analgesia for more than 48 hours. There were no side effects like motor block, nausea, vomiting, or respiratory depression. Continuous erector spinae plane block provides excellent analgesia in paediatric patients undergoing video-assisted thoracoscopic surgery, causing minimal side effects. Further, a prospective randomized control trial is suggested to establish the efficacy of this block in paediatric video-assisted thoracoscopic surgeries.

**Main Points**

- Video Assisted Thoracoscopic Surgery (VATS) is the treatment of choice in empyema in paediatric patients.
- There is very limited experience with continuous Erector Spinae Plane (ESP) block in paediatric age group.
- Ultrasonography (USG) guided ESP catheter insertion is a safe and effective way of providing prolonged analgesia in paediatric patients undergoing VATS.
- The index case series is a novel attempt where continuous ESP guided infusion has provided effective and prolonged analgesia in paediatric patients undergoing VATS.

## Introduction

Empyema thoracis continues to be a health burden in developing countries due to malnutrition, prevalence of tuberculous infection, delayed diagnosis of pneumonia, and delayed referral to higher centre.^1^

Decortication and stripping of infected pleura by video-assisted thoracoscopic surgery (VATS) or thoracotomy is the treatment of choice especially in Stage 2, that is, the fibrinopurulent stage. Video-assisted thoracoscopic surgery is supposed to have the advantages of a smaller incision, less postoperative pain, and a faster recovery as compared with thoracotomy.^2^ Stripping of pleura is associated with significant perioperative pain. The gold standard for the management is thoracic epidural analgesia; however, being a central neuraxial block, it is not without complications.^3^

Forero et al^4^ described the erector spinae plane (ESP) block in 2016. The experience of ESP block in paediatric patients is limited. We present our experience with continuous ESP block with a catheter, which includes 7 patients who underwent VATS and thus will help consolidate the overall experience with this novel block.

## Case Presentations

### Cases 1-5

These patients were cases of right-sided empyema who underwent VATS decortication. These were paediatric patients of age group 2-8 years of age, 4 females and 1 male. Before taking up the patient for induction, informed parental consent was taken for placement of a bilateral erector spinae catheter. At the induction of general anaesthesia, IV fentanyl 2 µg kg^−1^ iv was administered to all patients. Following this, the child was placed in the lateral position depending on the side of the surgery. After disinfection of the skin, counting downward from the C7 spinous process and using ultrasound guidance, the level of the T4 rib and the transverse process was identified. A high-frequency straight ultrasound (USG) probe was used (GE, Logiq^TM^ e, 4-10 MHz transducer), which was positioned transversely to visualise the right lateral tip of the T4 transverse process.

**Figure 1.**
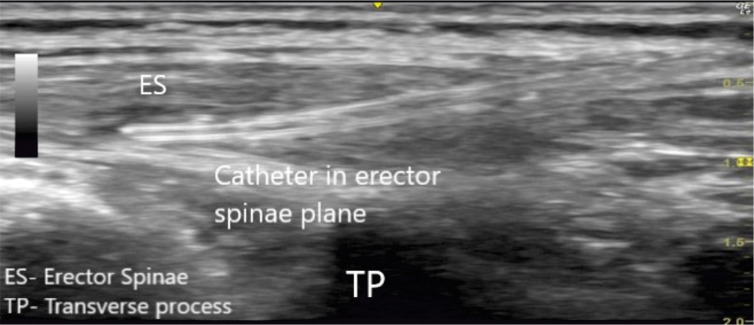
Catheter in the erector spinae plane which lies between erector spinae muscle above and the transverse process of vertebra below.

Using a B Braun Contiplex® D20 G needle, the ESP was identified between the transverse process and erector spinae muscle and confirmed by injecting 0.5-1 mL normal saline. A 20G 1000 mm catheter was then threaded into the space followed by injecting 0.3 mL kg^−1^ of 0.25% bupivacaine (Fig 1). The patient was taken back to supine position followed by the conduct of surgery. IV paracetamol 15 mg kg^−1^ slow iv infusion was given 30 minutes prior to the end of surgery. Postoperatively, the patients received infusion with bupivacaine and fentanyl up to 48 hours using a multidose elastomeric balloon pump (Baxter ® Multirate infuser). The rate of infusion was set at 0.25-0.3 mL kg^−1^ h^−1^ of a 0.125% bupivacaine solution + 1 µg mL^−1^ fentanyl. Analgesic efficacy during the surgery was assessed using surrogate markers like heart rate, blood pressure, end tidal carbondioxide (EtCO_2)__,_ temperature, and sweating. If there was a >10% rise in heart rate or blood pressure from baseline values in response to surgical stimuli, additional analgesic intravenous fentanyl 1 µg kg^−1^ was administered. Postoperative analgesia was assessed at 6, 12, 24, 36, and 48 hours using the Face, Legs, Activity, Cry, Consolability scale (0-10) for preverbal children, that is, up to 3 years and the Wong-Baker Faces Scale (0-10) for more than 3 years of age. IV diclofenac 1 mg kg^−1^ was used for postoperative rescue analgesia and rescue analgesic was given in case of pain scores more than 4/10. Safety was assessed by looking for adverse events in the form of motor blockade, nausea and vomiting, constipation, urinary retention, respiratory depression, toxicity of local anaesthetics, pruritus, and infection at the catheter site.

The surgical incision in all the cases was taken after 15-20 minutes of application of ESP block, and in this duration, monitoring of heart rate, blood pressure, saturation of pulsatile oxygen (SpO_2)_, EtCO_2_, and temperature was done and recorded every 5 minutes. There was no increase in these values by more than 10% during the incision in any of the patients. Hence, it was inferred that the block was effective. All the patients had excellent postoperative analgesia. The maximum pain score at 6 hours post-op was 2/10 and 0/10 at 24 hours and 0/10 at 48 hours. No adverse effects like motor block, nausea, vomiting, or respiratory depression were seen in any patient.

### Cases 6-7

These were 2 cases of left congenital diaphragmatic hernia aged 4.5 and 1.5 years, male and female, respectively, for thoracoscopic congenital diaphragmatic hernia (CDH) repair. After taking due informed consent from parents, the procedure for the application of the ESP block was the same as for Cases 1-5. In these cases, rescue analgesia was required in the 4.5-year-old child with a pain score 5/10 at 6 hours postoperatively. In the second case, single-shot ESP block had to be given (with 0.25% bupivacaine with 1 µg kg^−1^ clonidine) as the catheter could not be threaded. No adverse effects like motor block, nausea, vomiting, or respiratory depression were seen in any patient.

## Discussion

Erector spinae plane block is a new analgesic modality, especially in the paediatric population. Erector spinae plane block is performed by injecting an anaesthetic drug below the erector spinae muscle, i.e., in the inter‑fascial plane between this muscle and the transverse processes. The mechanism is partially due to local anaesthetic diffusing into the paravertebral space through the non-osseous spaces between adjacent vertebrae, thereby acting on both the dorsal and ventral branches of the thoracic spinal nerves^4,5^ as well as the communicating branches that feed the sympathetic chain.^4^ Because of the easy visualisation of the transverse process by USG and its safe distance from neuraxis and other vascular structures, it is a comparatively safe block to perform.^6^

In our experience with paediatric patients undergoing VATS procedures, this block provided excellent analgesia. Insertion of a catheter and use of continuous infusion of local anaesthetic and fentanyl successfully extended the period of analgesia for >48 hours.

## Conclusion

The experience of ESP blocks in paediatric patients undergoing VATS has been positive. This has shown excellent analgesia, high success rate, and minimal side effects.

## References

[1] Goyal V Kumar A Gupta M Sandhu HPS Dhir S . Empyema thoracis in children: still a challenge in developing countries. Afr J Paediatr Surg. 2014;11(3):206 210. (10.4103/0189-6725.137326) 25047309

[2] Mulder DS Pain management principles and anesthesia techniques for thoracoscopy. Ann Thorac Surg. 1993;56(3):630 632. (10.1016/0003-4975(93)90933-9) 8379756

[3] El-Morsy GZ El-Deeb A El-Desouky T Elsharkawy AA Elgamal MAF . Can thoracic paravertebral block replace thoracic epidural block in pediatric cardiac surgery: a randomized blinded study. Ann Card Anaesth. 2012;15(4):259 263. (10.4103/0971-9784.101848) 23041682

[4] Forero M Adhikary SD Lopez H Tsui C Chin KJ . The erector spinae plane block: a novel analgesic technique in thoracic neuropathic pain. Reg Anesth Pain Med. 2016;41(5):621 627. (10.1097/AAP.0000000000000451) 27501016

[5] Ueshima H Otake H . RETRACTED: Clinical experiences of ultrasound-guided erector spinae plane block for thoracic vertebra surgery. J Clin Anesth. 2017;38:137. (10.1016/j.jclinane.2016.12.028) 28372654

[6] Chong MA Berbenetz NM Lin C Singh S . Perineural versus intravenous dexamethasone as an adjuvant for peripheral nerve blocks: a systematic review and meta-analysis. Reg Anesth Pain Med. 2017;42(3):319 326. (10.1097/AAP.0000000000000571) 28252523

